# A deep learning pipeline for accurate and automated restoration, segmentation, and quantification of dendritic spines

**DOI:** 10.1016/j.crmeth.2025.101179

**Published:** 2025-09-18

**Authors:** Sergio Bernal-Garcia, Alexa P. Schlotter, Daniela B. Pereira, Aleksandra J. Recupero, Franck Polleux, Luke A. Hammond

**Affiliations:** 1 Department of Biological Sciences, Columbia University, New York, NY, USA; 2 Mortimer B. Zuckerman Mind Brain Behavior Institute, Columbia University, New York, NY, USA; 3 Department of Neuroscience, Columbia University, New York, NY, USA; 4 Champalimaud Research, Champalimaud Foundation, Lisbon, Portugal; 5 Department of Neurology, The Ohio State University Wexner Medical Center, Columbus, OH, USA

**Keywords:** deep learning, dendritic spines, neuronal morphology, image analysis, content-aware restoration, image segmentation, synaptic connectivity, fluorescence microscopy, open-source software, two-photon microscopy

## Abstract

Quantification of dendritic spines is essential for studying synaptic connectivity, yet most current approaches require manual adjustments or the combination of multiple software tools for optimal results. Here, we present restoration enhanced spine and neuron analysis (RESPAN), an open-source pipeline integrating state-of-the-art deep learning for image restoration, segmentation, and analysis in an easily deployable, user-friendly interface. Leveraging content-aware restoration to enhance signal, contrast, and isotropic resolution further enhances RESPAN’s robust detection of spines, dendritic branches, and soma across a wide variety of samples, including challenging datasets with limited signal, such as rapid volumetric imaging and *in vivo* two-photon microscopy. Extensive validation against expert annotations and comparison with other software demonstrate RESPAN’s superior accuracy and reproducibility across multiple imaging modalities. RESPAN offers significant improvements in usability over currently available approaches, streamlining and democratizing access to a combination of advanced capabilities through an accessible resource for the neuroscience community.

## Highlights

• RESPAN is user friendly and allows accurate and unbiased dendritic spine analysis
• RESPAN integrates deep learning restoration, segmentation, and extensive quantification
• RESPAN analyzes data with heterogeneous image quality without parameter tuning

## Motivation

Accurate and unbiased reconstructions of neuronal morphology and quantification of dendritic spines are widely used in neuroscience but remain a significant challenge for efficient large-scale analysis. Current methods rely heavily on manual annotation and parameter optimization between images, introducing bias and creating bottlenecks that limit large-scale studies. Additionally, existing automated tools often require complex workflows across multiple software platforms and lack integrated validation capabilities. We developed RESPAN to address these limitations by providing a comprehensive, automated pipeline that combines state-of-the-art deep learning approaches for image restoration and segmentation, model training, analysis, and validation within a single user-friendly graphical interface. This enables rapid, unbiased analysis of dendritic spine morphology across diverse imaging modalities while maintaining high accuracy and reproducibility.

## Graphical abstract

**Figure:**
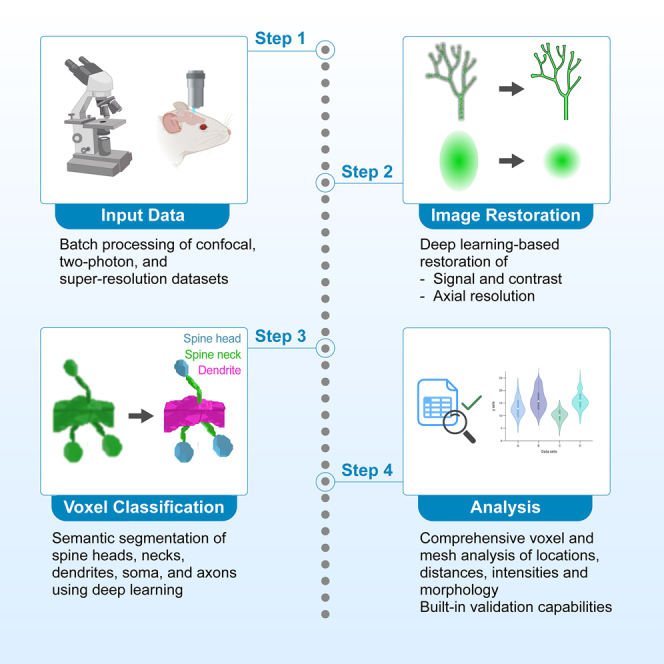

## Teaser

Garcia et al. present RESPAN, a pipeline that enables automated dendritic spine analysis through seamless integration of deep learning image restoration and segmentation capabilities with comprehensive quantitation. This streamlined approach ensures accurate and unbiased measurements, even under challenging imaging conditions. It also outperforms manual analysis and existing tools while democratizing access to a combination of advanced capabilities within a single application.

## Introduction

Dendritic morphology and synaptic input connectivity are among the cardinal features defining neuronal subtypes and their functional properties.^1^^,^^2^ In the mammalian neocortex, pyramidal neurons (PNs) receive the majority (>90%) of their excitatory synaptic inputs on dendritic spines, small protrusions first described by Santiago Ramón y Cajal.^3^^,^^4^ Analysis of dendritic spine density and distribution has been used for decades as a critical anatomical proxy for assessing excitatory input across different PN subtypes during development and in adult states, both in physiological and pathological contexts, including neurodevelopmental disorders and neurodegeneration.^5^^,^^6^^,^^7^ Furthermore, in adult cortical and hippocampal principal neurons, spine head size is tightly correlated with postsynaptic AMPA receptor content and synaptic strength^8^ (reviewed in Anggono and Huganir^9^ and Kessels and Malinow^10^). However, although this relationship can vary across neuronal populations,^11^ in developing neurons, spine size reflects a progressive increase in AMPA receptor content (increase in the AMPA/NMDA ratio), making spine size a key indicator of synaptic maturation.^12^

While recent deep learning (DL) approaches have improved the accuracy of dendritic spine mapping capabilities,^13^^,^^14^^,^^15^ their utility remains constrained by limited functionality and analysis readouts. Existing methods, such as Vaa3D, SpineTool, and Imaris,^1^^,^^16^^,^^17^^,^^18^^,^^19^^,^^20^ lack robustness and scalability across datasets and are highly sensitive to changes in signal and image quality. Consequently, full automation is rarely achieved, as each image often requires extensive manual intervention for parameter optimization and post-processing corrections. This reliance on manual adjustments limits efficiency and introduces human error and bias. While these tools can be combined with DL image restoration and segmentation tools, doing so requires significant technical expertise and creates complex, multi-step workflows that limit throughput and hinder broader adoption.

In recent decades, two key developments have made transformative advances in dendritic spine imaging. First, the introduction of bright fluorescent proteins combined with improved expression methods, including *in utero* electroporation (IUE), viral delivery, and transgenic mouse models, has enabled sparse, cell-type-specific labeling.^21^^,^^22^^,^^23^ Second, improvements in single- and multi-photon confocal microscopy have dramatically enhanced spatial and temporal resolution for both *in vitro* and *in vivo* imaging.^24^^,^^25^ However, accurately quantifying dendritic spine morphology and head volume, density, and spatial distribution remains challenging due to the reliance on labor-intensive manual or semi-automated reconstructions, which introduce subjectivity and constitute significant bottlenecks for large-scale connectivity studies.

To address these challenges, we developed restoration enhanced spine and neuron analysis (RESPAN), an integrated solution for the automated analysis of neurons and dendritic spines. RESPAN combines state-of-the-art DL approaches for content-aware image restoration, enhanced axial resolution, and robust 3D segmentation within a single user-friendly interface. The pipeline improves signal-to-noise, contrast, and spatial resolution before performing precise segmentation of spine heads, necks, dendritic branches, and soma. Unlike existing tools, RESPAN operates through an intuitive graphical user interface (GUI), eliminating coding requirements and seamlessly integrating multiple software environments to allow analysis, validation, and model training all within a single application. Additionally, RESPAN generates comprehensive readouts, including visual and tabulated data detailing dendritic spine morphology, signal intensity, and spatial metrics, as well as individual spine tracking for temporal analysis. We demonstrate RESPAN’s accuracy and robustness across multiple imaging modalities, representing a significant improvement in making advanced automated dendritic spine analysis accessible to the research community.

## Results

### Development of RESPAN

**Figure 1:**
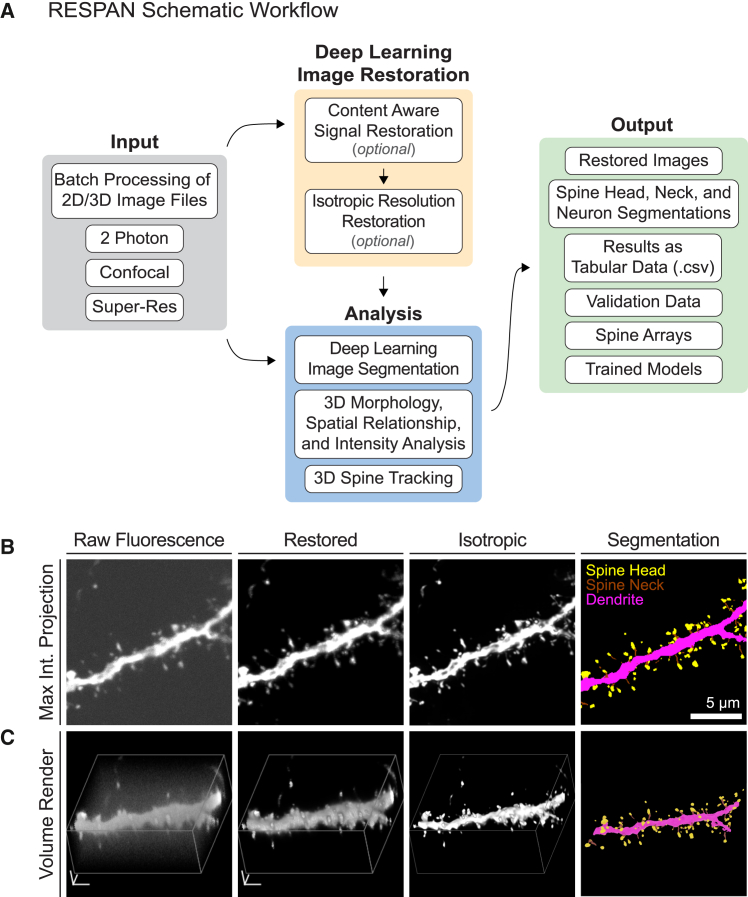
Overview of the RESPAN workflow for dendritic spine analysis (A) Schematic representation of the RESPAN workflow, beginning with the input of 2D/3D TIFF image files and progressing through multiple stages, including signal restoration, axial resolution restoration, deep learning image segmentation, and generation of quantitative outputs. RESPAN accommodates datasets from a variety of microscopy techniques, including two-photon, confocal, and super-resolution microscopy. (B) From left to right: examples of a raw fluorescence dataset acquired with a spinning disk confocal microscope (raw fluorescence), shown as a maximum intensity projection. Raw fluorescence images highlight reduced signals and resolution related to image acquisition. Following content-aware image restoration (restored), improvements in signal and contrast, essential for accurate spine detection, are apparent. Subsequent processing of the restored image to improve axial resolution reduces anisotropy (isotropic), thus improving the accuracy of morphological measurements. Finally, deep-learning-based segmentation on the restored image provides precise labels for dendrites, spine heads, and necks (segmentation). (C) 3D renderings of the same dataset shown in (B).

The RESPAN pipeline (Figure 1) integrates GPU image processing and multiple DL approaches to enable high-throughput fluorescent image restoration, segmentation, and analysis of dendritic branches and dendritic spines. By integrating content-aware restoration and 3D convolutional neural network segmentation, RESPAN improves spine detection accuracy and sensitivity to morphological variations, particularly in challenging experimental conditions such as *in vivo* two-photon microscopy and rapid volumetric imaging of large tissue volumes. To ensure broad accessibility, RESPAN is provided both as a Python code and as a standalone Windows application with an intuitive, unified GUI, allowing users to run batch analysis, train models, and perform analysis validation all within the same interface. Combining these features and multiple software environments within one interface substantially increases utility and reduces barriers for researchers without programming expertise.

### Step 1: DL image restoration (optional)

Prior to segmentation, RESPAN offers the option to perform image restoration to enhance signal quality and contrast. This is a critical step for accurate spine detection in challenging imaging conditions where phototoxicity and temporal resolution requirements limit image quality. To achieve this, RESPAN employs a 3D content-aware CSBDeep model,^26^ trained using paired low- and high-signal-to-noise ratio (SNR) image volumes. We acquired these image sets in various samples to best reflect the diversity of imaging conditions and biological features, including soma, dendritic branches, and spines, maximizing the generalizability of the trained model. This approach enables high-quality restoration that preserves important biological features. For spinning disk confocal microscopy, we used a set of six paired image volumes with dimensions of 1,500 × 1,500 × 130, a pixel size of 65 nm, and a Z-step size of 150 nm. Prior to training, the high-SNR datasets were processed using a blind deconvolution. For two-photon microscopy, we used 30 paired image volumes with average dimensions of ∼300 × 600 × 12, a pixel size of 102 nm, and a Z-step size of 1 μm. For both models, training was performed in Python using image augmentation (rotation, mirroring, and flipping) with patch sizes of 64 × 64 × 16 for 100 epochs, as previously described.^26^ Representative examples of CARE restoration applied to low-SNR datasets can be seen in Figure S1, illustrating how this approach can improve the fluorescence signal while preserving the morphological details needed for downstream spine segmentation.

Next, an optional axial restoration approach is used to reduce anisotropy and improve the accuracy of spine morphology measurements. This step is important for conventional microscopy techniques like confocal microscopy, where axial resolution is typically 2–3 times lower than lateral resolution. It becomes even more critical for two-photon microscopy, where the disparity between lateral and axial resolution can reach 4–5 times or more due to the elongated point spread function. These resolution differences significantly impact the accuracy of 3D measurements. To address such challenges, RESPAN incorporates Self-Net. This two-stage unsupervised neural network approach leverages the inherent anisotropy of microscopy data to improve axial resolution without requiring paired training data.^27^ By using lateral images as targets, Self-Net can learn to enhance axial resolution while maintaining high fidelity to genuine biological features. This approach necessitates models trained on image data that match the same specific experiment and imaging conditions for accurate results. To facilitate model training using user-specific datasets, we have integrated this capability within the RESPAN GUI, utilizing code adapted from the original Self-Net implementation.^27^

### Step 2: Image segmentation

For image segmentation, RESPAN employs a 3D full-resolution nnU-Net model, a self-configuring framework for biomedical image segmentation.^28^ This approach automatically optimizes network architecture and training parameters based on input data properties, ensuring robust performance across varied imaging conditions. To ensure broad utility, we created a diverse training dataset comprising 47 image volumes containing 2,489 expert-annotated spines from multiple imaging modalities commonly used in the field: Zeiss Airyscan confocal microscopy (63× 1.4 NA oil Plan Apochromat objective), Yokogawa W1 spinning disk confocal microscopy (100× 1.35 NA Nikon silicone Plan Apochromat objective), and Bergamo two-photon microscopy (25× 1.0 NA Olympus SCALEVIEW-A2 Plan objective).

In addition to modality-specific models, we pooled images from different modalities to create a generalizable high-resolution model. This involved standardizing the training data by resampling the data from the Airyscan confocal datasets to match the spinning disk confocal datasets with a voxel size of 65 × 65 × 150 nm. We expanded datasets through augmentation to further enhance model performance, including random rotations, flips, shot noise addition, and Gaussian blurring (Figure S2). This augmentation strategy improved model performance when combined with the on-the-fly patch augmentations generated by nnU-Net during training. Models were trained for 1,000 epochs with an initial learning rate of 0.01, following established parameters.^28^

Validation of our high-resolution global model against ground-truth (GT) labels demonstrated excellent performance, achieving Dice scores of 0.927 for spines, 0.986 for dendrites, and 0.991 for soma. Representative slices also show strong agreement between segmentation labels and corresponding features in raw fluorescence datasets, including spines with and without discernible necks (Figure S3). To maximize accessibility, RESPAN automatically scales input data to match model requirements while providing analysis output in the original resolution. This ensures that new users and novel datasets are likely to yield interpretable results without requiring additional preprocessing before using RESPAN. For cases where high-resolution resampling may be undesirable due to increased memory requirements, we recommend using a modality-specific model matching the acquisition resolution.

### Step 3: RESPAN output and quantification of dendritic spine morphometry

Following segmentation, RESPAN performs a comprehensive analysis of dendritic spines, providing numerical measurements for each spine (Table S1) and multiple validation outputs, including maximum intensity projections overlaying raw data with spine detections and distance measurements (Figure S4). Measurements include the volume of each spine, its centroid location, intensity measurements across all available channels, morphology statistics, and the distance from both the dendrite shaft and Euclidean and geodesic distances from the soma. Additional measurements, such as spine neck length and spine head width, are computed as previously described.^29^ Users can also specify constraints (e.g., minimum and maximum spine volume and distance from dendrite) to filter out spurious detections. Summary statistics include per-dendrite and per-image measurements of spine number, density, dendrite length, and average spine measurements (Table S2). RESPAN also supports the export of dendritic tracings for a single neuron or dendrite per image in standard SWC format via Vaa3D’s APP2 plugin.^30^ For rapid validation and data exploration, 2D and 3D arrays of all segmented spines, along with intermediate data (e.g., labeled segmentations, skeletons, and distance maps), can be saved, allowing users to verify accuracy and further refine segmentation models.

Importantly, RESPAN also includes a built-in validation module capable of automatically comparing results to GT annotations. This is essential for auditing RESPAN’s performance and for validating and reporting the accuracy of new restoration and segmentation models when publishing with RESPAN. The metrics provided by the validation module are described in Table S3.

### Comprehensive validation of RESPAN performance

We employed a comprehensive validation strategy combining both pixel-level and object-level metrics to evaluate RESPAN’s performance, following established practices in DL-based microscopy analysis.^22^^,^^24^ For pixel-level assessment, we measured the intersection over union (IoU) (|A ∩ B|/|A ∪ B|) and the Dice coefficient (DC) (2|A ∩ B|/|A| + |B|), where A represents RESPAN output pixels and B represents GT pixels. These metrics proved particularly valuable for assessing the precise delineation of spine and dendrite boundaries. For object-level validation, we assessed the accuracy of counting and characterizing individual spines by employing an IoU threshold of 50% to quantify successfully detected objects. The object-level comparison generated true positives (TPs), false positives (FPs), and false negatives (FNs), which we used to calculate two key metrics: the proportion of correctly detected objects (precision = TP/(TP + FP)) and the proportion of true objects that were correctly detected (recall = TP/(TP + FN)). Spine detection demonstrated excellent performance, achieving precision and recall scores of 0.988 and 0.9, respectively. We achieved a final F1 score of 0.94.

### Benchmarking RESPAN accuracy against human expert annotations

**Figure 2:**
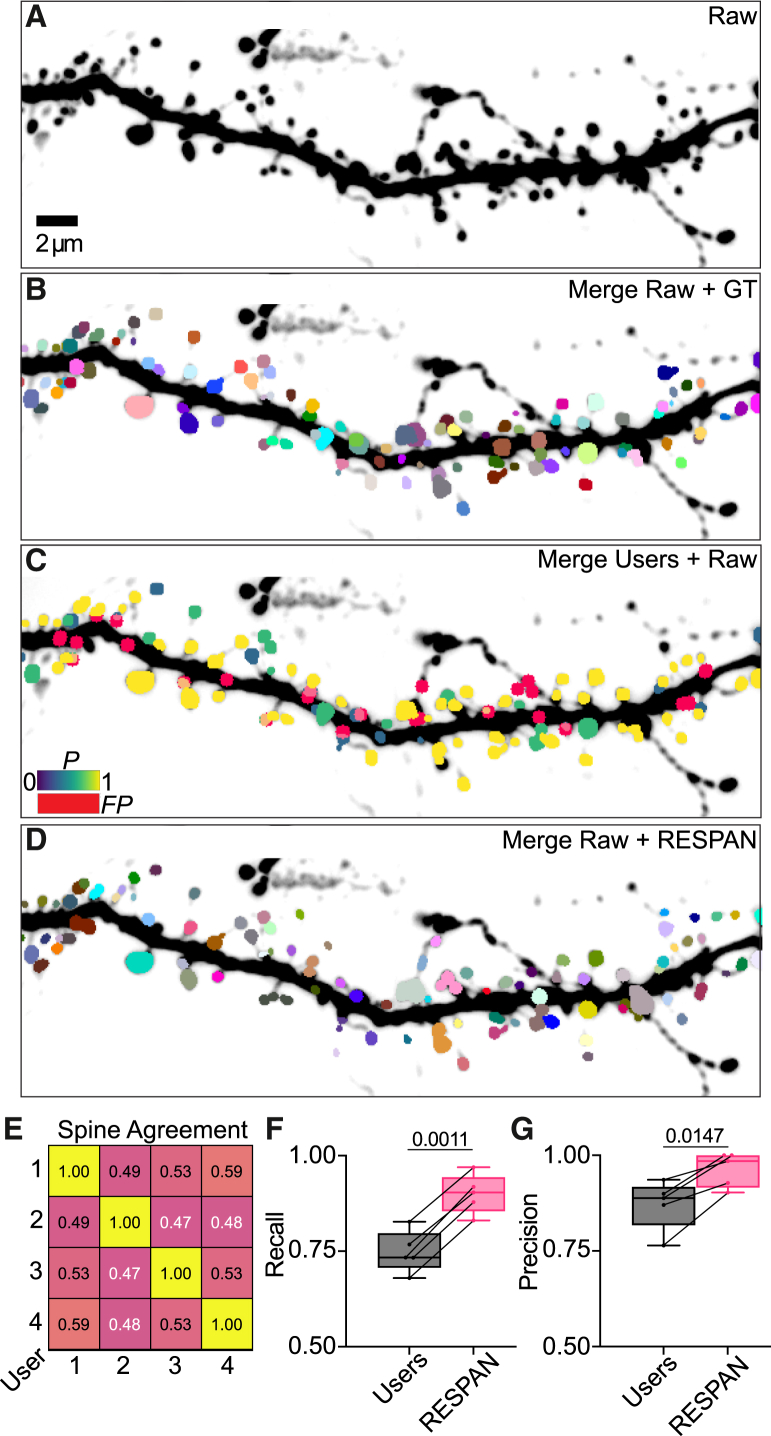
Evaluation of multi-user spine detection variability against RESPAN (A) Raw fluorescence image of a dendritic segment used as input for both manual annotation by multiple experts and automated processing by RESPAN. (B) Ground-truth (GT) annotations determined by a consensus of experienced researchers; colors reflect unique spines. (C) Composite image of the raw fluorescence with GT annotations overlaid. Spine colors reflect the probability of detection by four independent users, with false positive (FP) detections labeled in red. (D) Composite image merging raw fluorescence with RESPAN spine detections, with colors reflecting unique spines. (E) Inter-user correlation matrix showing the pairwise detection agreement among human annotators, with values ranging from 0 (no agreement) to 1 (perfect agreement). (F and G) Boxplots (median at center line, 5th–95th percentile range, and whiskers extending to the full data range) comparing recall and precision between human annotators and RESPAN, showing that RESPAN consistently achieves higher recall and precision, reflecting improved sensitivity and fewer FPs relative to manual human annotation. Pairwise detection agreement among human annotators (values from 0 to 1) was quantified using Pearson correlation. Recall and precision were compared between human annotators and RESPAN using a paired two-tailed t test (*n* = 5 pairs) at the 90% confidence interval. The resulting *p* values were *p* = 0.0011 for recall (F) and *p* = 0.0147 for precision (G). *p* < 0.05 was considered significant.

To assess the accuracy and reliability of RESPAN in detecting dendritic spines, we conducted a comprehensive comparison with manual annotations from multiple users of varying expertise (Figure 2). Using spinning disk confocal microscopy, we first acquired five independent fluorescence images of dendritic segments (Figure 2A). Two experts with years of experience in imaging and analyzing dendritic spines carefully annotated these images to create a GT consensus dataset (Figure 2B). We then used these GT datasets to evaluate spine detection performance against (1) four independent users with varying levels of expertise and (2) RESPAN output of the same images.

Each user analyzed the 3D raw fluorescence images in Fiji, marking putative spines at their perceived center of mass using the multi-point tool. We compiled the regions of interest (ROIs) placed by each user and superimposed them onto the GT consensus mask. We then used custom Python code to quantify the number of multi-point ROIs detected for each GT spine across all users and images, generating a spine detection probability map (Figure 2C). To determine inter-user agreement, we generated a correlation matrix depicting pairwise detection agreement among users, with values ranging from 0 (no agreement) to 1 (perfect agreement) (Figure 2E). This analysis highlighted known challenges in inter-experimenter variability and subjectivity in detecting spines, with poor agreement between users for spine detection and the potential for statistical biases in manual annotation approaches.

We then processed these same fluorescence images through the RESPAN pipeline for automated spine segmentation (Figure 2D). Using custom Python code, we then overlaid RESPAN’s segmentation output onto our GT consensus mask, employing an IoU criterion of 0.50 to determine correct spine detection. Comparing RESPAN’s performance to user detection probabilities revealed two key findings. First, RESPAN showed a marked decrease in missed detections compared to human annotators, more accurately identifying spines present in the GT consensus mask but missed by multiple users (Figure 2F). Second, RESPAN demonstrated improved detection of spines identified by only a subset of users, indicating superior sensitivity in challenging cases. Moreover, RESPAN showed a decrease in FPs when compared to non-expert annotators and was, therefore, more precise (Figure 2G).

These results demonstrate that RESPAN outperforms manual spine identification while alleviating inter-individual variability and human observer bias in image annotation. Furthermore, the automated approach provides comprehensive morphological readouts that are not feasible with human annotation, enabling a more thorough and unbiased analysis of dendritic spine populations.

### Demonstrating the importance of image restoration in spine detection

**Figure 3:**
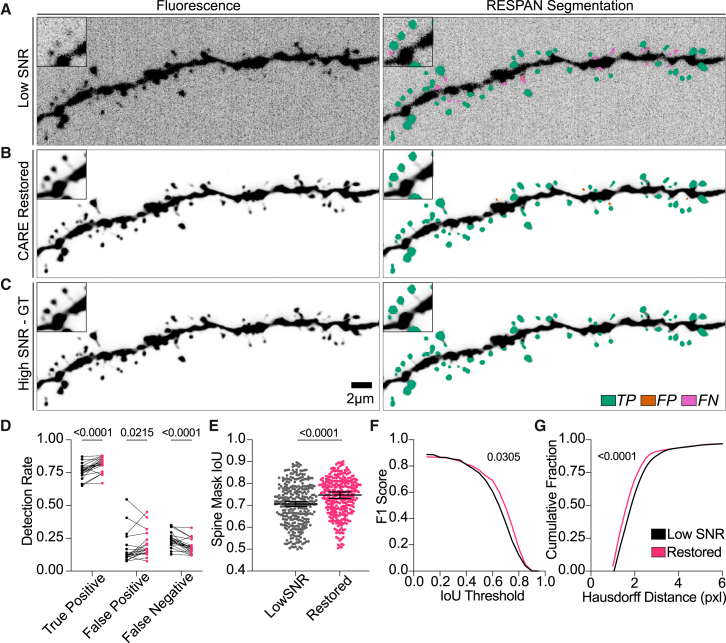
Importance of image restoration in spine detection accuracy (A–C) Maximum intensity projection images of a dendritic segment acquired under low-SNR conditions (A), following CARE restoration (B), and at a high SNR (C). The right images in each row show RESPAN’s segmentation outputs, with color-coded spines denoting true positives (TPs; green), false positives (FPs; orange), or false negatives (FNs; magenta). Insets highlight spines that are barely distinguishable in the low-SNR dataset but can be resolved following restoration. (D) Detection rates (TPs, FPs, and FNs) for low-SNR (black) vs. restored (magenta) datasets. Statistical comparisons were performed using Wilcoxon matched-pairs signed-rank tests with a two-stage step-up method to control the false discovery rate. (E) Spine mask intersection-over-union (IoU = 0.5) analysis comparing segmentation labels from low-SNR and restored images to those from the high-SNR output. Each point represents a matched spine, and the line/whiskers depict the median and 95% confidence interval (CI); *p* values were obtained using Wilcoxon matched-pairs signed-rank tests. (F) F1 scores plotted across increasing IoU thresholds (0.1–0.9). Comparisons between low-SNR (black) and restored (magenta) curves were performed using Wilcoxon matched-pairs signed-rank tests. (G) Cumulative distributions of Hausdorff distances for low-SNR (black) and restored (magenta) spines, where lower values indicate closer alignment with the ground-truth spine shape. Data were analyzed by a Kolmogorov-Smirnov test, revealing significantly reduced Hausdorff distances in restored data.

We next investigated how content-aware restoration could improve RESPAN’s segmentation performance in low-SNR imaging scenarios commonly encountered in live or *in vivo* two-photon experiments (Figure 3). Although our global model 1 already provides reasonable segmentation on low-SNR datasets, our results indicate that applying a CARE restoration model^26^ prior to RESPAN processing further enhances spine detection and segmentation metrics for low-SNR data (Figures 3D–3G). Notably, in this study, we intentionally used extremely low-SNR images to reflect “worst-case” imaging conditions. Even under these stringent conditions, the detection of spines with an IoU ≥ 0.5 rose from a median of 0.70–0.75 (Figure 3E), and the fraction of TP spines increased from 0.76 to 0.82 (Figure 3D). Furthermore, the F1 score, which captures both recall and precision, showed a marked increase in its cumulative distribution as the IoU threshold became more stringent (Figure 3F), reflecting the improved accuracy when segmenting restored datasets.

We additionally assessed the boundary accuracy using the Hausdorff distance (Figure 3G), which measures how far the most extreme mismatched boundary point lies from GT surfaces. After CARE restoration, we observed significantly lower Hausdorff distances, indicating that restored spines were closer to their GT counterparts. This improvement is crucial for downstream measurements of spine volume or surface area, where a single poorly placed boundary point could skew morphological calculations.

Equally important, the ability to reliably restore low-SNR data has practical benefits for experimental design. By capturing images with shorter exposure times and reduced laser power, users can increase throughput while mitigating photobleaching and phototoxicity. In turn, the restored images effectively regain high-SNR features, ensuring accurate quantification. Thus, our workflow enables high-fidelity segmentation of spines with efficient and gentle acquisition parameters, which is particularly valuable for long time-lapse series or large-volume acquisitions, where preserving sample health and reducing acquisition time are paramount.

### Biological validation of RESPAN using established phenotypes affecting spine density and morphology

**Figure 4:**
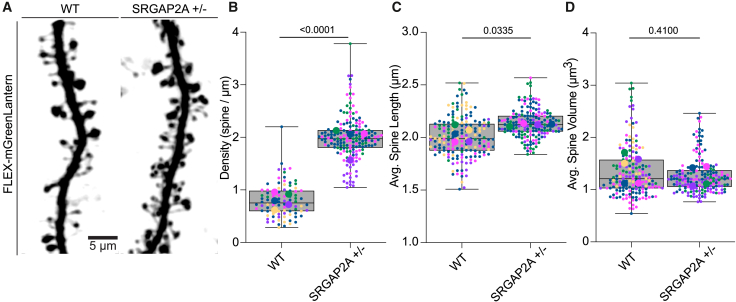
Blind genotype/phenotype validation using RESPAN (A) *In vivo* two-photon images acquired through chronic cranial windows of sparsely labeled layer 2/3 dendrites from wild-type (WT) and *SRGAP2A*^+/−^ mice. (B) Spine density (spines/μm) is significantly higher in *SRGAP2A*^+/−^ dendrites than in WT (nested t test, ∗∗∗∗*p* < 0.0001). (C) Mean spine length is modestly but significantly increased in *SRGAP2A*^+/−^ dendrites (nested t test, ∗*p* = 0.0335). (D) Mean spine volume does not differ between genotypes (nested t test, n.s., *p* = 0.4100). For all plots, data are nested (dendrites within animals) and displayed as box plots (center line, median; box 5th–95th percentile; whiskers, full range). Dendrites from the same animal share the same color. Larger outlined circles represent mean values for each animal. Sample sizes: 91 dendritic segments from five WT animals and 179 dendritic segments from four *SRGAP2A*^+/−^ animals.

We then sought to validate RESPAN’s ability to detect well-established phenotypes in adult layer 2/3 PNs from *SRGAP2*^+/−^ mice, namely, increased spine density and increased spine neck length, but there was no significant change in spine volume distribution compared to adult wild-type (WT) littermates.^1^^,^^31^^,^^32^^,^^33^^,^^34^ We performed a blinded genotype/phenotype analysis using *in vivo* two-photon microscopy through a cortical window of dendritic segments from layer 2/3 PNs of live WT and *SRGAP2*^+/−^ knockout mice (Figure 4A). For this analysis, neurons were sparsely labeled via IUE with a plasmid FLEX-mGreenLantern together with a low amount of another plasmid expressing Cre-recombinase.^35^

RESPAN analysis successfully replicated these established phenotypes through an unbiased and blinded approach. Quantitative analysis revealed that *SRGAP2*^+/−^ mutants displayed significantly higher spine density than WT neurons (Figure 4B, nested t test, ∗∗∗∗*p* < 0.0001), confirming the expected increase.^1^^,^^31^^,^^32^^,^^33^^,^^34^ Similarly, spines in the *SRGAP2*^+/−^ mutants showed an increase in mean spine length when compared to WT spines (Figure 4C, nested t test, ∗*p* = 0.0335), but there was no significant difference in spine volume between WT and *SRGAP2*^+/−^ mice (Figure 4D, nested t test, n.s., *p* = 0.4100), consistent with our previous findings using manual analysis. This validation demonstrates RESPAN’s ability to accurately detect and quantify dendritic spine phenotypes in an unbiased manner, providing results that directly align with previously published evidence.

**Figure 5:**
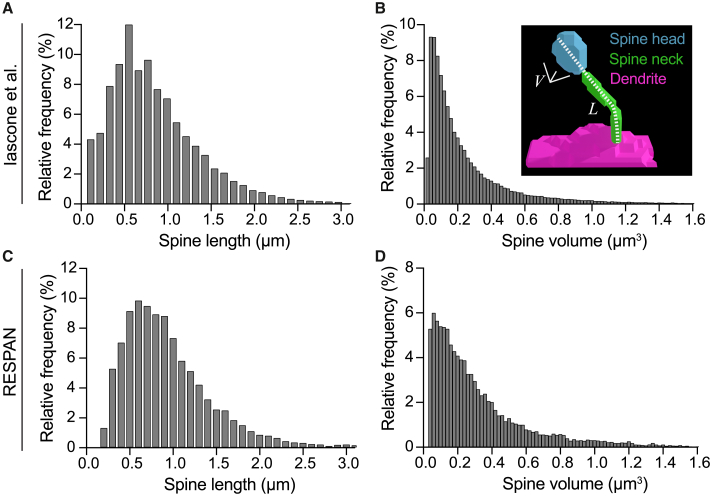
Spine lengths and volumes measured by RESPAN match known distributions (A and B) Histogram displaying the relative frequency distribution of spine lengths (A) and spine volumes (B) in wild-type (WT) neurons, based on a previous analysis of 81,604 spines, serving as the ground truth (GT). The *x* axes represent spine length (μm) and volume (μm^3^), with the relative frequency (%) of spines within each bin noted on the *y* axes. The inset in (B) provides an example of a segmented dendritic spine, color coded to illustrate the spine head (blue), spine neck (green), dendrite (magenta), spine head volume (V), and spine length (L). (C and D) Histograms of spine length (C) and volume (D) distributions in WT neurons based on RESPAN analysis of 10,945 spines, revealing a high degree of similarity in distributions compared to GT data.

To further validate RESPAN’s accuracy against established metrics, we analyzed spine morphology distributions in a larger population of WT PNs and compared these results to previously published GT data from multiple isolated PNs where all excitatory and inhibitory spines were mapped and analyzed (Figures 5A and 5B).^1^ This analysis of 81,604 spines revealed characteristic distributions: spine length following a normal distribution and spine volume showing a Poisson distribution with predominant small spines and a smaller population of large spines. Using RESPAN, we analyzed 10,945 spines from multiple WT dendritic segments imaged by spinning disk confocal microscopy, using a minimum and a maximum spine volume threshold of 0.035 and 2 μm^3^, respectively. RESPAN analysis resulted in distributions for spine length and volume with comparable distributions to those previously published (Figures 5C and 5D), demonstrating that RESPAN consistently replicates established biological distribution patterns for spine length and volume.

### Spine tracking

**Figure 6:**
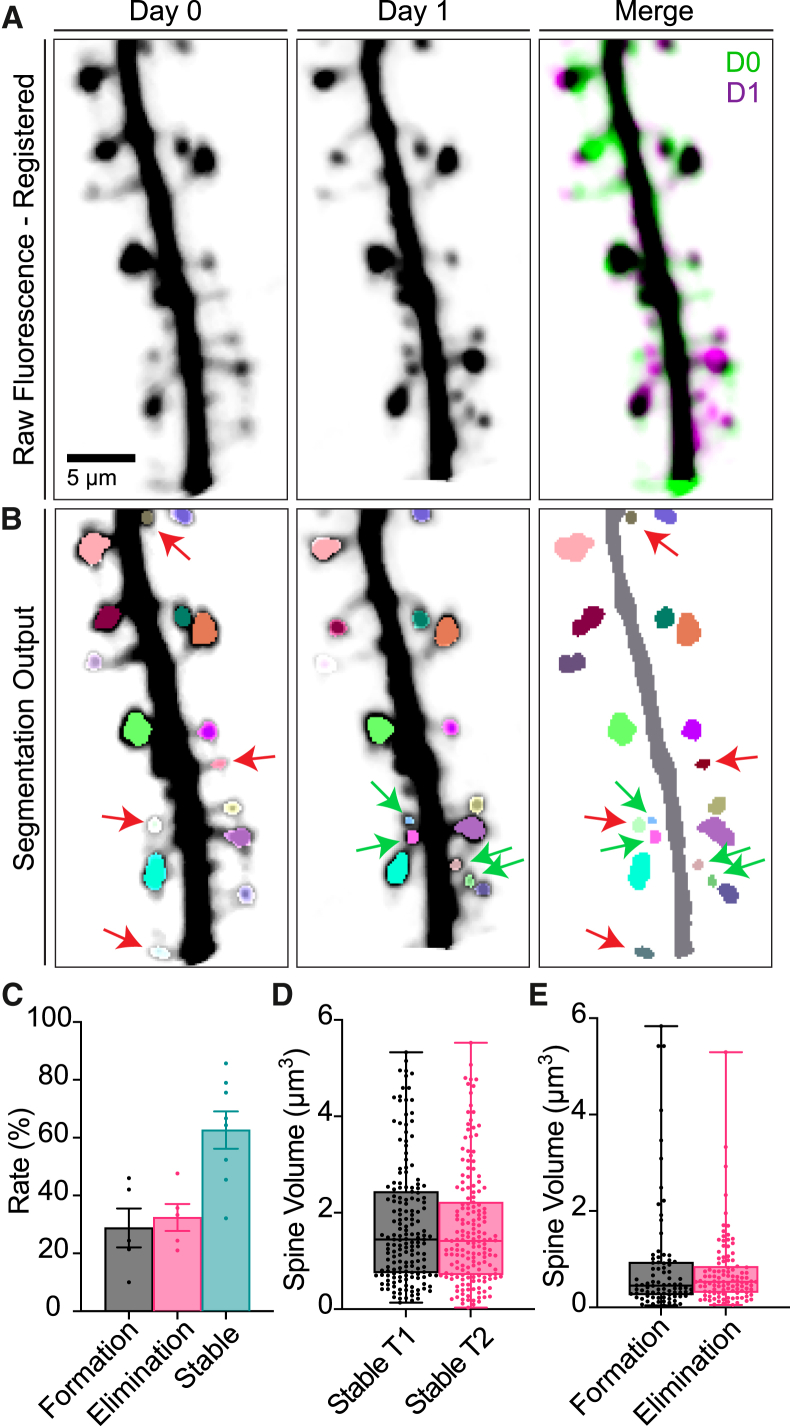
Application of RESPAN for the analysis and tracking of spines *in vivo* (A) Representative two-photon microscopy images showing dendritic spines on days 0 (left) and 1 (center), with their merged visualization (right). Raw fluorescence data were registered between time points to enable precise spine tracking. (B) Segmentation output from RESPAN, demonstrating accurate identification of individual spines across time. Stable spines detected at both time points are shown in the same color. Newly formed spines are indicated by green arrows, while eliminated spines are marked by red arrows at their respective time points. (C) Quantification of spine dynamics showing the rate (%) of stable spines from day 0 to 1 and the relative proportions of spine formation and elimination events. Data are presented as bar plots with mean ± SEM. (D) Volume comparison between stable spines at both time points. (E) Distribution of spine volumes for newly formed and eliminated spines. Boxplots show median, quartiles, and min/max distribution of individual measurements. Sample size: 279 spines from 8 dendritic segments. No statistical difference was observed for (D) and (E).

Maintaining a consistent mapping of dendritic spines across multiple time points is a key challenge in longitudinal spine analysis, particularly under *in vivo* conditions where brain tissue may shift or deform. To address this, RESPAN provides a comprehensive spine tracking feature that integrates 3D volumetric registration, segmentation, and label matching into a single workflow. As depicted in Figure 6, we applied this method to two separate high-resolution two-photon z stacks obtained via a cranial window in a living mouse. RESPAN begins by allowing the user to perform either rigid or non-rigid 3D motion correction on each stack, ensuring that corresponding dendritic structures align accurately across sessions.

After registration, the pipeline segments dendrites and spines and tracks them across time, maintaining unique spine IDs. Spines that persist between sessions share the same ID, whereas newly formed and eliminated spines are labeled distinctly (Figures 6A and 6B). This color-coded visualization enables rapid identification of formation, elimination, and stabilization events (Figures 6C–6E). Metrics evaluated during temporal analysis are documented in Table S4.

A unique strength of RESPAN is the seamless integration of volumetric alignment and tracking capabilities with restoration and segmentation, streamlining analysis and eliminating the need for specialized coding or computational expertise. This allows researchers to process folders containing multiple imaging sessions with their desired motion-correction method and readily verify alignment quality. To our knowledge, this marks the first GUI-based pipeline that combines 3D motion correction, restoration, segmentation, and longitudinal spine tracking in an integrated, semi-automated manner. By simplifying these previously complex workflows, RESPAN enhances the practical feasibility of analyzing *in vivo* spine dynamics, allowing for a quantitative assessment of changes in spine number and morphology over time.

### Advantages and comparison with other methods

**Figure 7:**
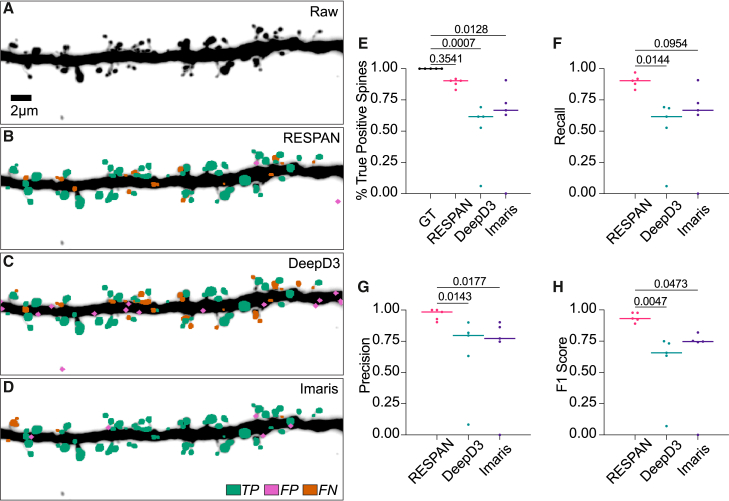
Comparison of RESPAN performance with other software (A) Maximum intensity projection of a raw fluorescence dataset showing a dendritic segment. (B–D) Spine detection outputs from RESPAN (B), DeepD3 (C), and Imaris (D) overlaying the dendritic segment. Detected spines are color coded as true positives (TPs; green), false positives (FPs; magenta), and false negatives (FNs; orange). (E) Percentage of TP spines detected by each method. (F) Recall scores for spine detection, with RESPAN demonstrating consistently higher recall, reflecting fewer FNs. (G) Precision scores for spine detection, with RESPAN outperforming DeepD3 and Imaris by reducing FP detections. (H) F1 scores for spine detection, representing the harmonic mean of precision and recall. RESPAN achieves superior F1 scores compared to other methods. (E)–(H) show a solid line at the median. Sample size: 440 GT spines from 11 dendritic segments.

RESPAN addresses critical challenges of reproducibility and efficiency in spine analysis, outperforming existing tools in both ease of use and accuracy (Figure 7). To benchmark its performance, we directly compared RESPAN’s spine detection results with those of DeepD3, a DL-based spine detection tool, and Imaris, a widely used commercial solution for 3D image analysis. The data used for this comparison comprised image volumes acquired using typical imaging parameters that reflected diverse image quality related to protein expression, depth, and position of the dendrites within the tissue sections. For each software tool, optimal parameters were determined and kept constant across all datasets. Fine-tuning of parameters between datasets would likely yield better results for Imaris and DeepD3, but this strategy prevents high-throughput analysis and increases the risk of bias. Both DeepD3 and Imaris exhibit higher rates of FPs (magenta) and FNs (orange) than RESPAN (Figures 7A–7D). This reduced accuracy reflects the sensitivity of these approaches to variability in image quality and a reliance on parameter tuning. We next benchmarked performance in cases of minimum SNRs, representing extremely low signals and challenging imaging conditions, and ideal SNRs, acquired using high laser powers impractical for typical acquisitions and long integration times with frame averaging followed by deconvolution. This comparison confirmed that quantification using Imaris can be highly accurate when the SNR is high and consistent between images but that performance is significantly reduced for datasets with mixed or low SNRs (Figure S5). While RESPAN outperformed DeepD3, we observed that DeepD3 was successful in accurately detecting spine and dendrite voxels; however, the detection of spine objects remained lower, despite sweeping through the available spine detection parameters in the DeepD3 interface.

Quantitative metrics confirm that RESPAN achieves superior recall (*p* = 0.0144 vs. DeepD3; *p* = 0.0954 vs. Imaris), precision (*p* = 0.0143 vs. DeepD3; *p* = 0.0177 vs. Imaris), and F1 score (*p* = 0.0047 vs. DeepD3; *p* = 0.0473 vs. Imaris) (Figure 7H). The percentage of TP spines detected by RESPAN also remains consistently higher than that of the other tools, reflecting more robust performance across different dendritic morphologies. These findings highlight RESPAN’s strengths as a fully automated, broadly applicable approach that is less sensitive to image-to-image variability and minimizes the need for manual calibration or correction.

For a broader overview of how RESPAN compares to other methods, please refer to Table S5. While many commonly used workflows combine multiple software programs or rely on significant manual intervention, RESPAN integrates state-of-the-art DL modules for restoration, segmentation, and validation into a single GUI. This integration substantially reduces technical barriers and promotes reproducibility, enabling researchers to audit results and retrain models for new datasets with ease.

To our knowledge, RESPAN is the only freely available tool that integrates multiple state-of-the-art DL approaches—content-aware image restoration,^26^ axial resolution enhancement,^27^ and self-configuring image segmentation.^28^ While recent advances have shown the power of individual DL approaches for specific tasks, such as DeepD3 for spine detection or CARE and Self-Net for image restoration, combining these capabilities requires significant computational expertise and manual data handling between different software tools and environments. RESPAN addresses this limitation by providing a comprehensive, easily deployed, and integrated solution, eliminating the technical barriers and overhead typically associated with using multiple separate analysis tools. Additionally, instead of performing image operations on the CPU, RESPAN leverages GPU processing to efficiently handle images in parallel, including the rapid generation of 2D and 3D spine arrays.

Critically, unlike other methods, RESPAN provides a built-in tool for automatically validating the quality of analysis against GT data. This addresses an unmet need, as segmentation validation is often performed manually or less rigorously by researchers with limited coding experience. By incorporating this capability, RESPAN can encourage a rigorous approach to automated quantification and increase the reproducibility of published results. RESPAN also facilitates training new restoration and segmentation models directly within the GUI, which would otherwise require separate Python environments and coding experience, significantly reducing the barriers to adopting this approach.

RESPAN significantly lowers the barriers to performing advanced image quantification for researchers without coding expertise. This addresses challenges presented by other tools that require a combination of Python or MATLAB scripts without a GUI, including tools that have been incorporated into our approach. Furthermore, unlike other methods that limit analysis outputs to dendritic spine coordinates or require the use of multiple programs in different environments,^13^ our approach provides comprehensive 3D analysis within a single GUI environment. RESPAN can also be readily adapted to new research questions by correcting initial results from the provided models using Fiji,^36^ a widely used image analysis software, and the tools within RESPAN.

## Discussion

Dendritic spine analysis remains a cornerstone for understanding neuronal connectivity. Yet, current approaches, predominantly manual or semi-automated, are constrained by interactive annotation steps, image-specific parameters, user-dependent variability, and limited throughput. In addressing these challenges, RESPAN introduces a fully integrated, DL-enhanced solution that significantly broadens the scope and reliability of automated spine analysis.

One of RESPAN’s significant contributions is its integrated architecture, which consolidates image restoration, axial resolution enhancement, and DL-based 3D segmentation into a single application. This design minimizes the biases inherent in manual and conventional thresholding methods, facilitating reproducible and direct comparisons across diverse imaging modalities. With an increasing focus on methodological rigor, RESPAN provides a streamlined and robust framework for quantifying synaptic plasticity, neuronal development, and disease models.

RESPAN also addresses significant barriers related to accessibility. Harnessing state-of-the-art computational image analysis has historically required specialized expertise or costly commercial platforms. By integrating multiple software environments, GPU acceleration, model training, and analysis validation into a user-friendly interface, RESPAN lowers these barriers and democratizes the use of advanced analysis tools. This broad accessibility has the potential to accelerate discoveries in fields ranging from basic synaptic physiology to translational research on neurodevelopmental disorders and neurodegenerative diseases, areas where subtle changes in spine morphology and distribution may reveal profound shifts in neuronal function.

It is important to note that the morphological metrics provided by RESPAN, and by comparable methods, are constrained by the resolution limits of light microscopy, with electron microscopy (EM) being the gold standard for precise measures of morphology and spine classification.^37^^,^^38^ However, recent advancements in expansion microscopy protocols now enable ∼20 nm resolution on conventional confocal microscopes, increasing the accessibility of ultrastructural imaging.^39^ The approaches employed by RESPAN are readily compatible with such datasets. However, the substantially larger file sizes associated with these experiments may necessitate adjustments to the workflow and longer processing times.

Looking forward, RESPAN’s architecture provides a platform for future extensions. Further integration of additional segmentation models and analysis tailored to related biological features, such as mitochondria and organelle analysis along dendrites and in relation to spines, as well as pre- and postsynaptic protein analysis, will further enhance its utility.

A key aim in developing RESPAN was to democratize access to an advanced and complex computational pipeline in an open-source, easy-to-deploy platform. RESPAN is publicly available, and we are committed to maintaining this status to ensure equitable access for all research groups, independent of financial resources. Releasing RESPAN under an open-source license also enables the broader neuroscience community to actively contribute to its ongoing development, thereby enhancing its robustness, adaptability, and longevity. We anticipate that future community-driven improvements will extend RESPAN’s applicability to additional imaging modalities and progressively optimize computational efficiency in response to emerging advances in hardware and software technologies.

In conclusion, RESPAN represents a significant advancement in automated dendritic spine analysis, delivering accurate, robust, and accessible quantification to the neuroscience community. By enabling unbiased, high-throughput measurements of spine morphology and distribution, RESPAN paves the way for more comprehensive and reproducible studies of neuronal connectivity. Furthermore, by sharing the restoration and segmentation models along with the code as an open-source platform, RESPAN fosters collaboration and iterative development, allowing it to evolve alongside the research community’s needs and ensuring its sustained relevance and impact.

### Limitations of the study

While RESPAN addresses numerous challenges in automated dendritic spine quantification, it does have limitations. Notably, its performance is heavily contingent on DL models trained with datasets that accurately label the biological features being quantified. Similarly, image restoration models perform poorly when applied to datasets acquired using hardware and parameters that differ from those used in their training data. While RESPAN includes tools for model retraining, future iterations could benefit from more comprehensive, pre-trained models that cover a wider range of imaging conditions.

RESPAN currently supports input data exclusively in TIFF format, requiring the conversion of proprietary formats to TIFF before use. To address this limitation, we provide a Fiji macro to facilitate batch conversion to TIFF via OMERO Bio-Formats.^40^ We also generate our output data in TIFF for ease of use, but future releases would benefit from incorporating support for the OME-Zarr format to improve scalability.^41^

Users should be aware of the challenges associated with applying DL models to microscopy data.^26^^,^^42^ Content-aware restoration and axial enhancement models should ideally be trained on data that match the biological features, resolution, and imaging modality of the intended application. When models are applied to data that are significantly different from their training set, they may perform sub-optimally and introduce artifacts by hallucinating features or degrading genuine structures. This is particularly relevant when comparing experimental conditions with substantial morphological differences. In such cases, training data should encompass the full range of expected biological variation to ensure reliable results. Furthermore, as demonstrated in recent studies,^27^ the performance of these models can degrade when the resolution anisotropy ratio exceeds 4-fold or when the input data have extremely low SNRs. To address these limitations, RESPAN includes model validation and retraining tools, allowing users to optimize performance for their specific experimental conditions.

An important consideration for interpreting spine measurements from RESPAN, or any other fluorescence microscopy-based method, is the limitation imposed by diffraction-limited resolution. Although advanced microscopy techniques, such as Airyscan (as used here), approach super-resolution (∼100 nm lateral resolution), they do not achieve the nanoscale accuracy (∼10–20 nm) available through EM, which remains the gold standard for ultrastructural quantification of dendritic spines. Direct comparison between spine dimensions obtained via RESPAN and EM-based measurements is not feasible due to this fundamental resolution gap. Thus, RESPAN does not aim to provide EM-equivalent resolution or absolute nanoscale accuracy. Instead, RESPAN is designed to deliver consistent, reliable quantification and validation within the optical resolution limits typical of fluorescence microscopy, enabling unbiased, high-throughput analysis in routine experimental settings. Users should thus exercise caution when interpreting absolute spine dimensions from any fluorescence-based quantification method. Encouragingly, with ongoing advances such as expansion microscopy, it may become increasingly feasible to combine RESPAN’s analytic robustness with improved optical resolution, further narrowing the gap between optical and EM-based measurements in future studies. Moreover, previous research has demonstrated that direct quantitative comparisons between neuronal morphological measurements acquired via EM and light microscopy are inherently unreliable due to fundamental methodological differences in resolution and staining methods. Specifically, light microscopy consistently underestimates or misrepresents critical structural features such as dendritic branching complexity, spine volume, and synapse morphology compared to EM.^37^^,^^38^^,^^43^^,^^44^

Another important consideration relates to computational resources required for running RESPAN, some of which scale according to the size of the data being analyzed. We have made concerted efforts to optimize computational efficiency by integrating GPU acceleration and data chunking strategies to substantially reduce resource demands. Nevertheless, minimum hardware specifications are required for effective use, primarily an NVIDIA GPU, which is a common requirement for DL software and remains beyond our control. While most research institutions typically have access to computational workstations sufficient for RESPAN’s requirements, ensuring universal compatibility with all available hardware configurations is inherently challenging. By carefully balancing computational performance and accessibility, we believe RESPAN effectively reduces the resource-related barriers inherent to advanced microscopy analysis software, although certain practical constraints inevitably remain.

## Resource availability

### Lead contact

Requests for further information and resources should be directed to and will be fulfilled by the lead contact, Luke A. Hammond (luke.hammond@osumc.edu).

### Materials availability

No new materials or reagents were generated in this study.

### Data and code availability

• Original raw data in TIFF format acquired using spinning disk and two-photon confocal microscopy, along with results generated by RESPAN, have been deposited on Mendeley at Mendeley Data: https://data.mendeley.com/datasets/hdvsw62w5r/2 and are publicly available as of the date of publication.
• All original code and a standalone Windows application are publicly available on Zenodo at Zenodo Data:https://doi.org/10.5281/zenodo.15612240 as of the date of publication. These can also be accessed at https://github.com/lahammond/respan.
• Any additional information required to reanalyze the data reported in this work paper is available from the lead contact upon request.

## Acknowledgments

We thank Emiliano Zamponi from the Polleux lab for helping with image annotation and the Polleux lab members for extensive discussions and annotations to generate the multi-user metrics. We thank Qiaolian Lu for her help with mouse colony management. Imaging was performed in the Zuckerman Institute’s Cellular Imaging platform. BioRender.com assets were used in the graphical abstract. We also gratefully acknowledge use of the research computing resources of the Empire AI Consortium, Inc., with support from the Empire State Development of the State of New York, the Simons Foundation, and the Secunda Family Foundation. This work was supported by internal funding from the Zuckerman Institute, the NIH (5U19NS104649 and R35 NS127232), the NOMIS Foundation, and the FCT (EXPL/MED-NEU/0279/2021 and CEECIND/02245/2017).

## Author contributions

Conceptualization, S.B.G., F.P., and L.A.H.; methodology development, S.B.G., A.P.S., and L.A.H.; data acquisition and formal analysis, S.B.G., A.P.S., D.B.P., A.J.R., and L.A.H.; investigation, S.B.G., F.P., and L.A.H.; writing – original draft, S.B.G. and L.A.H.; writing – review & editing, S.B.G., D.B.P., F.P., and L.A.H.; funding acquisition, F.P.; resources, F.P. and L.A.H.; supervision, F.P. and L.A.H.

## Declaration of interests

The authors declare no competing interests.

## STAR★Methods

### Key resources table

**Table:** 

REAGENT or RESOURCE	SOURCE	IDENTIFIER
**Chemicals, peptides, and recombinant proteins**		

ProLong™ Glass Antifade Mountant	ThermoFisher	Cat#: P36982
Citifluor™ Mountant Solution MWL4-88	EMS	Cat#: 17977-150
Citifluor™ Mountant Solution AF100	EMS	Cat#: AF100-5

**Deposited data**		

Examples Datasets and Results	This study	*https://data.mendeley.com/datasets/hdvsw62w5r/2*

**Experimental models: Organisms/strains**		

Mouse: C57BL/6J	Jackson Laboratories	RRID:IMSR_JAX:000664
Mouse: SRGAP2A ±	Charrier. et al.^31^	N/A

**Recombinant DNA**		

pCAG-Cre	Matsuda et al.^45^	Addgene plasmid #13775
pEf1α-FLEX-tdTomato	Iascone et al.^1^	N/A
pEF1ɑ-FLEX-mGreenLantern	Cambell et al.^46^	Addgene plasmid #164468

**Software and algorithms**		

RESPAN (Restoration Enhanced SPine And Neuron Analysis)	This study	https://doi.org/10.5281/zenodo.15612240
nnU-Net	Isensee et al.^28^	N/A
CARE	Weigert et al.^26^	N/A
DeepD3	Fernholz et al.^13^	N/A
Fiji	ImageJ	https://imagej.net/software/fiji/
StackReg plugin	BIG-EPFL	https://bigwww.epfl.ch/thevenaz/stackreg/
NIS Elements Deconvolution Module	NIS-Elements	https://www.microscope.healthcare.nikon.com/en_EU/products/software/nis-elements
GraphPad Prism 10.4.1	Prism	https://www.graphpad.com/
Imaris	Oxford Instruments	https://imaris.oxinst.com/
Self-Net	Ning et al.^27^	N/A

**Other**		

3D printed headplate for cranial windows	Zuckerman Institute Advanced Instrumentation	N/A
Yokogawa W1 spinning disk confocal	Yokogawa Electric Corporation	N/A
Bergamo II 2-photon microscope	Thorlabs	N/A

### Experimental model and study participant details

#### Animals and experimental design

All animal procedures were approved by the Institutional Animal Care and Use Committee (IACUC) at Columbia University and conducted in accordance with institutional and federal animal welfare guidelines (IACUC Protocols AC-AABL2557 and AC-AABV8659). Mice were housed in a temperature-controlled environment on a 12-h light/dark cycle, with *ad libitum* access to food and water.

#### In vivo imaging

Species/strain: C57BL/6J (B6) and constitutive heterozygous knockouts for SRGAP2A^+/−^

Genotype: WT (B6) and SRGAP2A^+/−^ Age/Developmental Stage: Mice were implanted with cranial windows at postnatal day (P)90 and imaged between P100–P120.

Sex: Both male and female mice were used; sex was recorded for all animals.

Housing and Maintenance: Standard housing conditions; individually housed post-surgery to prevent injury.

Ethical Oversight: All procedures were approved by Columbia University IACUC.

#### Ex vivo imaging

Species/Strain: C57BL/6J (B6)

Genotype: WT.

Age/Developmental Stage: Neurons were labeled *in utero* at embryonic day (E)15.5, with brains harvested at postnatal stages.

Sex: Both male and female embryos were used; sex was not a variable in the analysis.

Housing and Maintenance: Pregnant dams were housed under standard conditions until embryos reached E15.5 for *in utero* electroporation.

Ethical Oversight: Procedures were approved by Columbia University IACUC.

#### Sex as a biological variable

Both male and female mice were used in this study. While sex was recorded for all animals, the data were analyzed without sex-based segregation, as prior research indicates no significant sex-dependent differences in the measured parameters. However, potential sex-related effects are acknowledged as a limitation of this study.

### Method details

#### Animal handling and surgical procedures

For *in vivo* imaging, mice underwent cranial window implantation at P90 under isoflurane anesthesia. A 4 mm circular craniotomy was performed over primary somatosensory cortex (S1), and a glass coverslip (No. 1, 4 mm diameter) was affixed with dental cement. A custom 3D printed headplate was attached to facilitate head fixation during imaging. Postoperative analgesia included subcutaneous injections of meloxicam (5 mg/kg). Mice were allowed 7–14 days for recovery before imaging.

For *ex vivo* experiments, *in utero* electroporation was performed at E15.5 to label Layer 2/3 pyramidal cortical neurons with fluorescent plasmids. Pregnant dams were anesthetized with isoflurane (1.5–2%), and embryos were injected with a plasmid mixture containing pCAG-Cre (1–10 ng/μL) and pCAG-FLEX-TdTomato (1 μg/μL). Plasmids were delivered into the lateral ventricle, followed by electroporation using five 50 ms pulses at 35 V (BTX ECM830 electroporator, 1 Hz frequency). Pups were allowed to develop postnatally before brains were extracted for imaging.

#### Tissue preparation for ex vivo imaging

Mice were anesthetized with isoflurane and transcardially perfused with ice-cold phosphate-buffered saline (PBS) followed by 4% paraformaldehyde (PFA) in PBS. Brains were dissected and post-fixed in 4% PFA overnight at 4°C, then rinsed in PBS and sectioned at 100 μm using a Leica VT1000 S vibratome. Sections were mounted in ProLong Gold antifade reagent for imaging.

#### In vivo two-photon imaging

Imaging was performed using a Bergamo II two-photon microscope (Thorlabs) equipped with a Ti:Sapphire laser (Coherent Chameleon Ultra II, 920 nm excitation). A 25 ×1.0 NA water-immersion objective (Olympus, XLPlan N) was used for data acquisition. Image stacks were acquired at 1024 × 512 pixels with a pixel size of 0.102 μm and 1 μm axial step size. The laser operated at an 80 MHz repetition rate with a 100 fs pulse width, and power at the sample was maintained at <15 mW to minimize phototoxicity. Mice were head-fixed on a custom stage, and imaging sessions lasted <60 min per session to prevent physiological stress, minimize photodamage, and minimize exposure to anesthetic.

#### Spinning disk confocal imaging (ex vivo samples)

Sections were imaged using a Yokogawa W1 spinning disk confocal system mounted on a Nikon Ti2 inverted microscope controlled with NIS-Elements. Images were acquired with a 100 ×1.35 NA SR HP Plan Apo silicone immersion objective and a Prime BSI sCMOS camera (Teledyne Photometerics), resulting in a pixel size of 65nm and a z-step size of 150nm. 488 and 561 nm laser lines were used to sequentially excite GFP and TdTomato, respectively, with matched bandpass emission filters installed in a triggered high-speed filter wheel (IDEX Health & Science LLC). When appropriate, images were further processed using a blind deconvolution algorithm with 25 iterations using the low noise parameter in the NIS Elements.

#### Data acquisition and processing

*In vivo* time-lapse imaging was performed via daily imaging over two continuous days to track dendritic spine dynamics across a 24-h interval. *Ex vivo* datasets were processed for 3D reconstruction and dendritic spine analysis using RESPAN (Restoration Enhanced SPine and Neuron Analysis), which integrates content-aware image restoration with deep learning-based segmentation. Motion correction for *in vivo* imaging was conducted using StackReg (https://github.com/fiji-BIG/StackReg) to compensate for minor movement artifacts. Spine detection was performed using nnU-Net-based segmentation models, which were trained on a diverse dataset of manually annotated spines to ensure robustness across varying imaging conditions. Quantifications included spine density, spine head volume, and spine-to-dendrite distance, with statistical validation performed against expert-annotated ground truth datasets.

#### Content-aware restoration model training

For CARE model training, paired datasets reflecting low and ideal SNR conditions were acquired on a Yokogawa W1 spinning disk confocal equipped with a 100×1.45 NA Plan Apochromat objective. Low-SNR datasets were acquired using a 488 nm laser at ∼0.3 mW and a 30 msec exposure time, while ideal or high-SNR datasets used ∼6 mW and a 400 msec exposure time with 2-fold averaging and 2-fold integration. A subset of these paired datasets was withheld when training the CARE restoration model and subsequently used for evaluating restoration performance.

#### nnU-net model training

To train our Global Model 1, we used nnU-Net v2.4 on a single H100 GPU instance from the Lambda GPU Cloud. We used the Lambda Stack environment, which includes PyTorch and other dependencies for nnU-Net already compiled, enabling straightforward deployment on x86_64 architectures. Notable improvement in segmentation accuracy was observed by increasing the training batch size to 19 and the patch size to [32, 256, 256]. The Python environment and dependencies on Ubuntu 22.04 LTS we managed using Lambda Stack’s preconfigured packages to streamline setup and execution. All relevant installation commands and hardware configuration details are provided in our public repository to promote reproducibility on multiple hardware platforms.

### Quantification and statistical analysis

All statistical analyses were performed using GraphPad Prism 9.0. Statistical details for each experiment, including sample sizes (n), measures of central tendency, variability, and statistical significance, are reported in the figure legends and corresponding text in the Results section.

#### Statistical tests and data distribution

To compare differences in dendritic spine density, spine length, and volume between WT and SRGAP2A^+/−^ mice, we used Kolmogorov-Smirnov tests, a non-parametric method suitable for comparing distributions without assuming normality. For comparisons of imaging-based segmentation performance (e.g., spine detection accuracy, precision, recall, and F1 score), we employed paired two-tailed t-tests and Wilcoxon matched-pairs signed-rank tests, as appropriate. For datasets involving multiple comparisons, two-way ANOVA followed by Benjamini-Krieger-Yekutieli correction was applied to control the false discovery rate (FDR).

#### Definition of sample size (n)

For *in vivo* imaging, each n represents an individual dendritic segment imaged from a single neuron, and multiple neurons were imaged per animal. For *ex vivo* experiments, each n corresponds to an apical dendritic segment from a single neuron; multiple neurons were imaged from multiple mice. Final sample sizes were based on prior studies assessing dendritic spine morphology in similar experimental paradigms and are reported in figure legends.

#### Randomization and blinding

Mice were randomly assigned to experimental groups at the time of imaging, and all spine analyses were performed blinded to genotype to reduce bias. Image processing and quantification via RESPAN also remained blind to the genotype to minimize manual intervention and ensure unbiased analysis.

#### Image segmentation and analysis

Analysis in Imaris used an autopath approach with manual soma position and seed placements to ensure accurate dendrite tracing. Spine detection used a thinnest diameter setting of 0.3 μm and a maximum length of 5 μm. The seed point threshold was then adjusted to a value of 140 to ensure maximum spine detection whilst avoiding false positives. For DeepD3, the following ROI-building parameters in the GUI were selected for a representative image: ROI building method = connected components; area threshold = 0.1; peak threshold = 0.1; minimum planes per ROI = 1; minimum 3D ROI size = 10 px; maximum 3D ROI size = 10 000 px; maximum prediction difference to seed pixel = 0.2; maximum 3D Euclidean distance to seed pixel = 2 px; watershed disabled. These values were selected to be as inclusive as possible and were then applied unchanged to the entire dataset using the provided DeepD3 batch-processing script. Prior to inference, the imaging volumes were resampled in XY from a native resolution of 65 nm/pixel to 94 nm/pixel to match the resolution of the best-performing DeepD3 model (“DeepD3_32F_94nm”). After batch processing with the parameters described above, the DeepD3 outputs were resampled back to 65 nm/pixel in XY before quantitative comparison with the ground-truth spine annotations. Because DeepD3 performs inference plane-by-plane, no scaling was necessary along the z axis.

#### Segmentation validation and accuracy metrics

RESPAN’s automated segmentation performance was validated against ground truth expert annotations, and performance was quantified using Dice coefficient, F1 score, precision-recall curves, and Hausdorff distance. Object-level segmentation accuracy was assessed by comparing IoU (Intersection over Union) values with a threshold of 0.50 to define correctly detected spines.

#### Statistical significance criteria

For all analyses, statistical significance was defined as *p* < 0.05, with exact *p*-values reported in the figures and results section. Data distributions were examined for normality, and appropriate parametric or non-parametric tests were applied based on distribution characteristics.

## Supplemental information

Document S1. Figures S1–S5 and Tables S1–S5

Document S2. Article plus supplemental information
