# Supplementary material for: A deep learning pipeline for accurate and automated restoration, segmentation, and quantification of dendritic spines
Source: Cell Rep Methods. 2025 Sep 18;5(10):101179. doi: 10.1016/j.crmeth.2025.101179 (PMC12570329; doi:10.1016/j.crmeth.2025.101179)
Supplement: Document S1. Figures S1–S5 and Tables S1–S5 [file mmc1.pdf]

Cell Reports Methods, Volume *5*

Supplemental information

A deep learning pipeline for accurate and automated restoration, segmentation, and quantification of dendritic spines

Sergio Bernal-Garcia, Alexa P. Schlotter, Daniela B. Pereira, Aleksandra J. Recupero, Franck Polleux, and Luke A. Hammond

Supplementary Figures

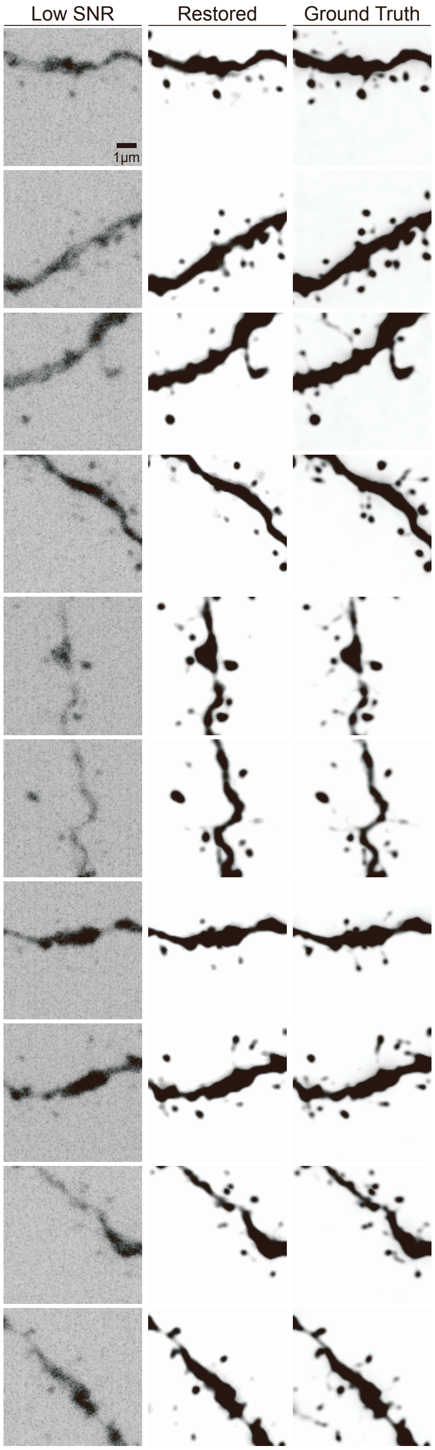

Figure S1. Representative examples of CARE restoration. Related to Figure 3.

Isolated maximum intensity projection views of a dendritic segment are shown under three conditions: low-SNR acquisition (left), low-SNR CARE-restored output (middle), and high-SNR ground truth data (right). Content-aware restoration of signal and contrast reveals spine heads, necks, and dendritic features that are difficult to discern in low-SNR images. Scale bar, 1 μm.

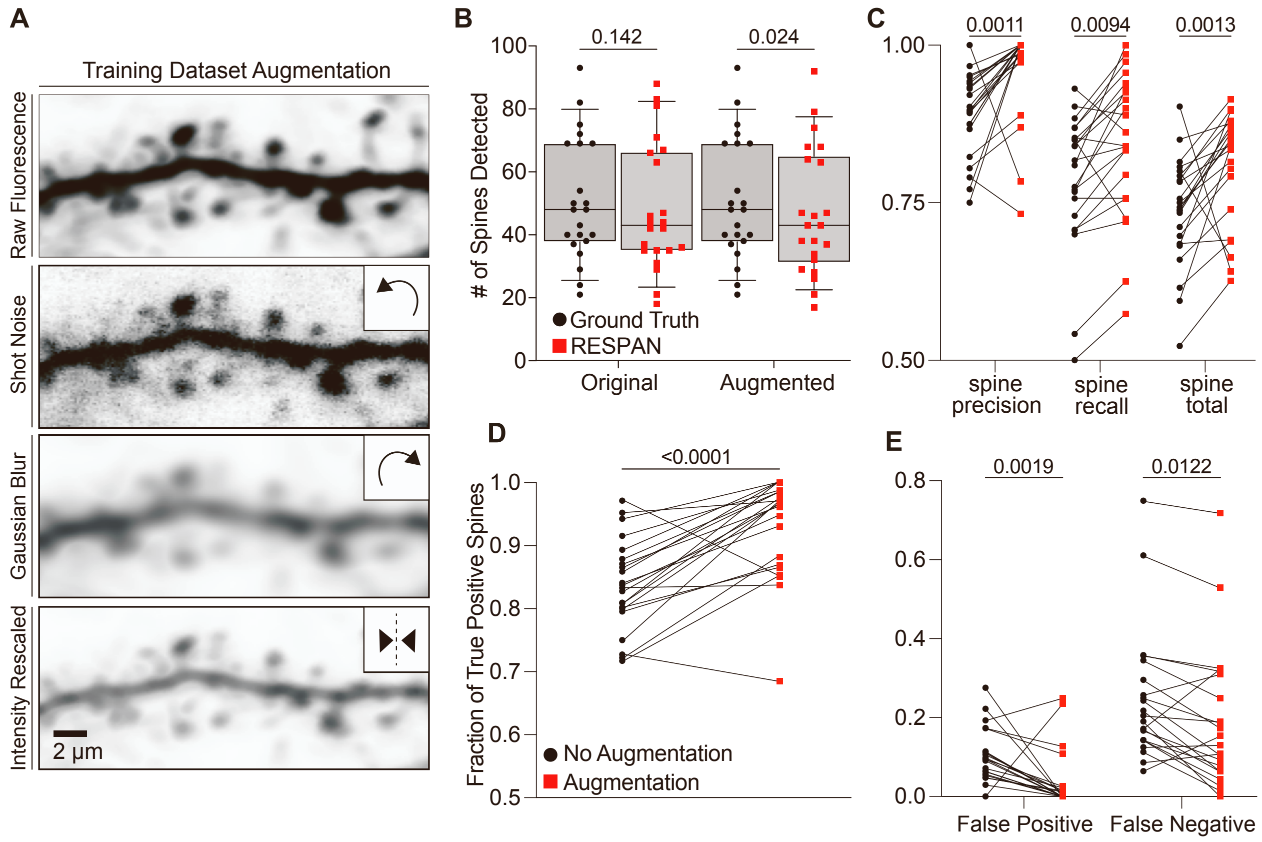

Figure S2. Enhancement of spine detection performance through data augmentation. Related to STAR Methods.

(A) Data augmentation techniques applied during model training, illustrated through representative images of original raw fluorescence and subsequent transformations including shot noise, Gaussian blur, and pixel intensity scaling, aimed at enhancing model robustness and generalizability. Random rotations and flips were also performed during augmentation, expending the dataset for model training. Scale bar, 2 μm. (B) Comparison of the number of raw spine counts detected in original and augmented datasets by ground truth (black) and RESPAN (red), analyzed using two-way ANOVA, indicating no significant difference (ns) to significant differences in detection counts. (C) Scatter plots comparing spine detection metrics, precision, recall, and total performance between original and augmented datasets, analyzed using two-way ANOVA with statistical significance indicated (**$p < 0.01$). (D) Bar graph showing the fraction of true positive spines detected, comparing models trained with no augmentation (black) and with augmentation (red) indicating a statistically significant increase in the detection of true positives, analyzed using the Wilcoxon matched-pairs signed-rank test (****$p < 0.0001$). (E) Scatter plot illustrating the reduction in false positives and false negatives in augmented datasets, analyzed using two-way ANOVA, with statistical tests indicating significant differences in error rates (**$p < 0.01$, *$p < 0.05$).

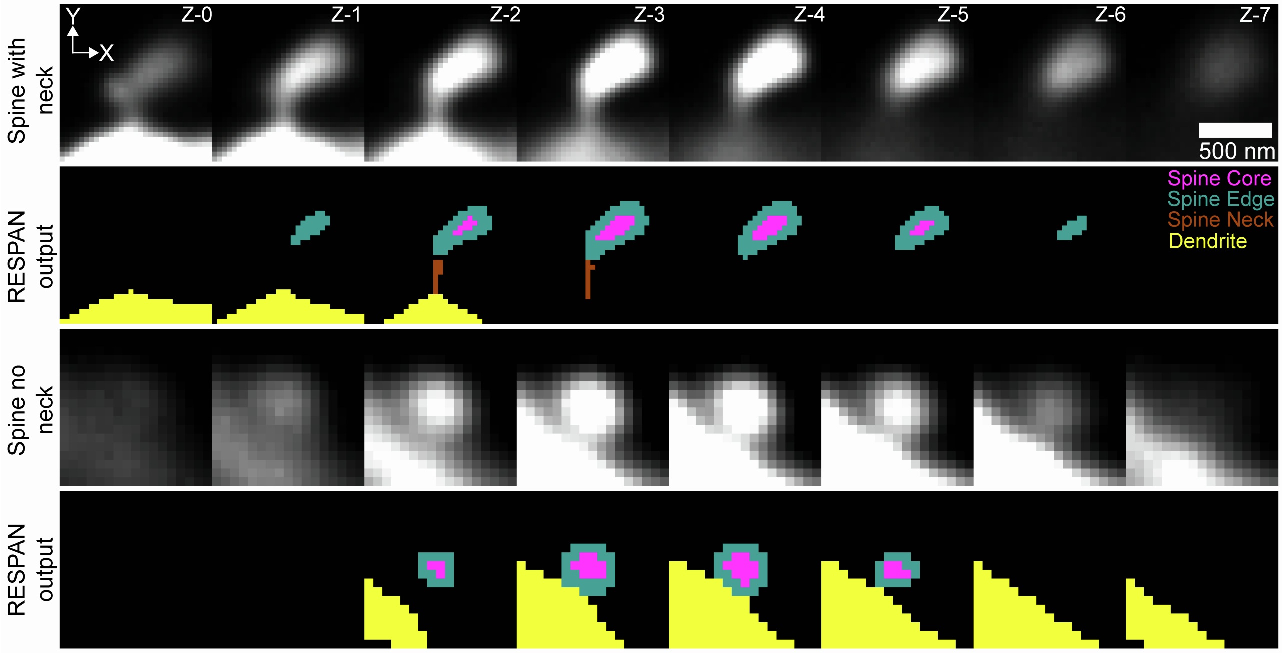

Figure S3. RESPAN reliably detects dendritic spines with and without discernible necks. Related to STAR Methods.

(A) Cropped slices from a raw fluorescence volume showing a representative dendritic spine possessing a clearly discernible neck. (B) RESPAN segmentation of the same spine, color-coded to show spine head core (magenta), spine head shell (teal), spine neck (brown), and dendrite (yellow), confirming correct detection of the narrow neck. (C) Cropped slices from a raw fluorescence volume showing a spine head directly connected to the dendritic shaft and lacking a discernable neck. (D) RESPAN segmentation of this spine correctly classifies the spine core, spine edge and dendrite while confirming the absence of a neck. Scale bar, 500 nm.

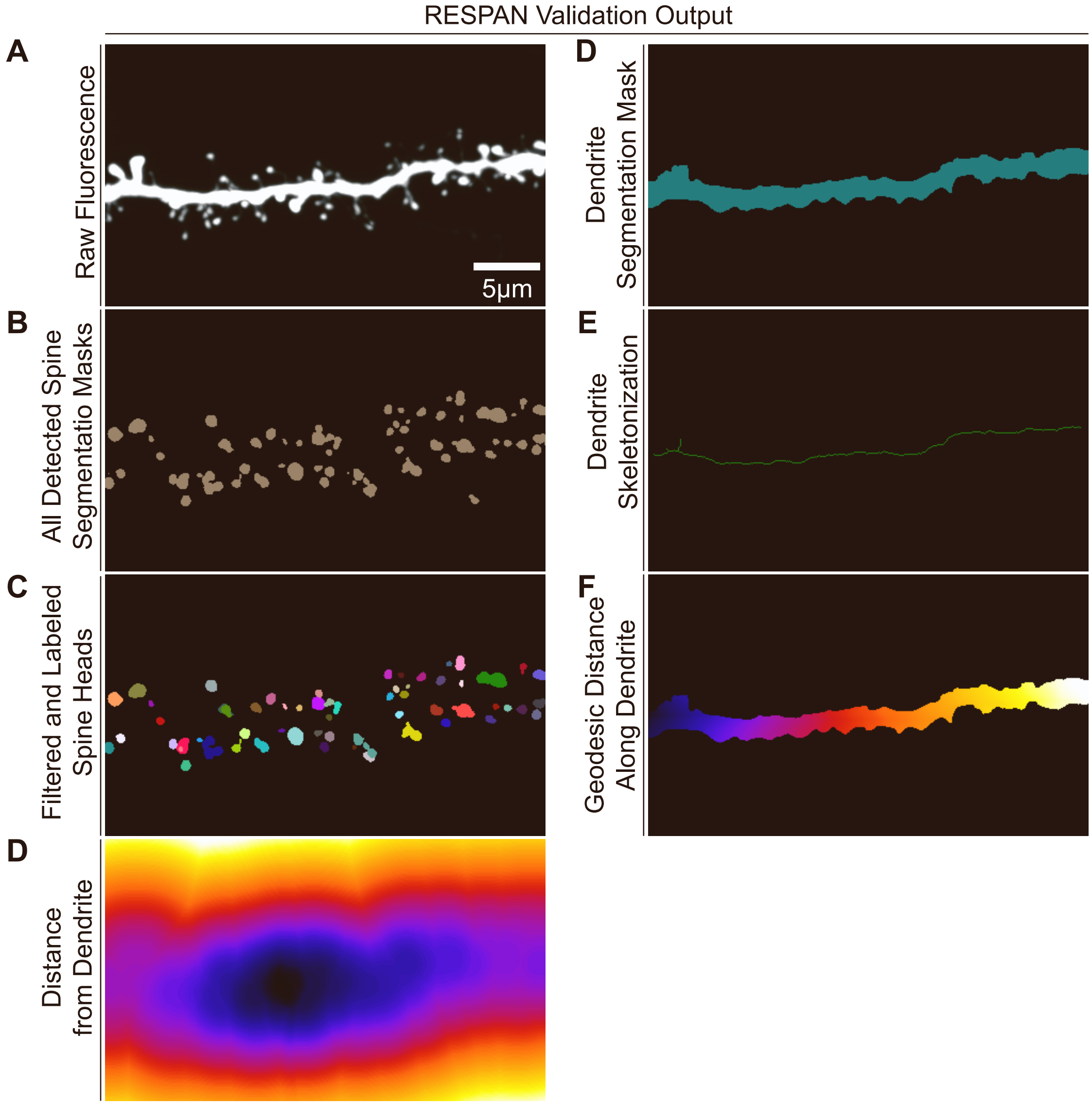

Figure S4. RESPAN validation output for dendritic spine detection and analysis. Related to STAR Methods.

Maximum intensity projection views automatically generated by RESPAN for validating analysis. (A) Raw fluorescence image of the analyzed dendritic segment. (B) All putative spine segmentation masks. (C) Uniquely labeled spine segmentation masks after applying morphological criteria (minimum/maximum volume; maximum distance from dendrite). (D) Heatmap representing each voxel's distance from the dendrite shaft (cooler hues, closer distance; warmer hues, greater distance). (F) Corresponding dendrite segmentation mask. (E) Dendrite skeleton. (F) Color-coded geodesic distance computed along the dendrite in 3D (blue to white, proximal to distal). These validation outputs allow users to readily confirm accurate segmentation of dendritic spines and the parent dendrite. Scale bar, 5 µm.

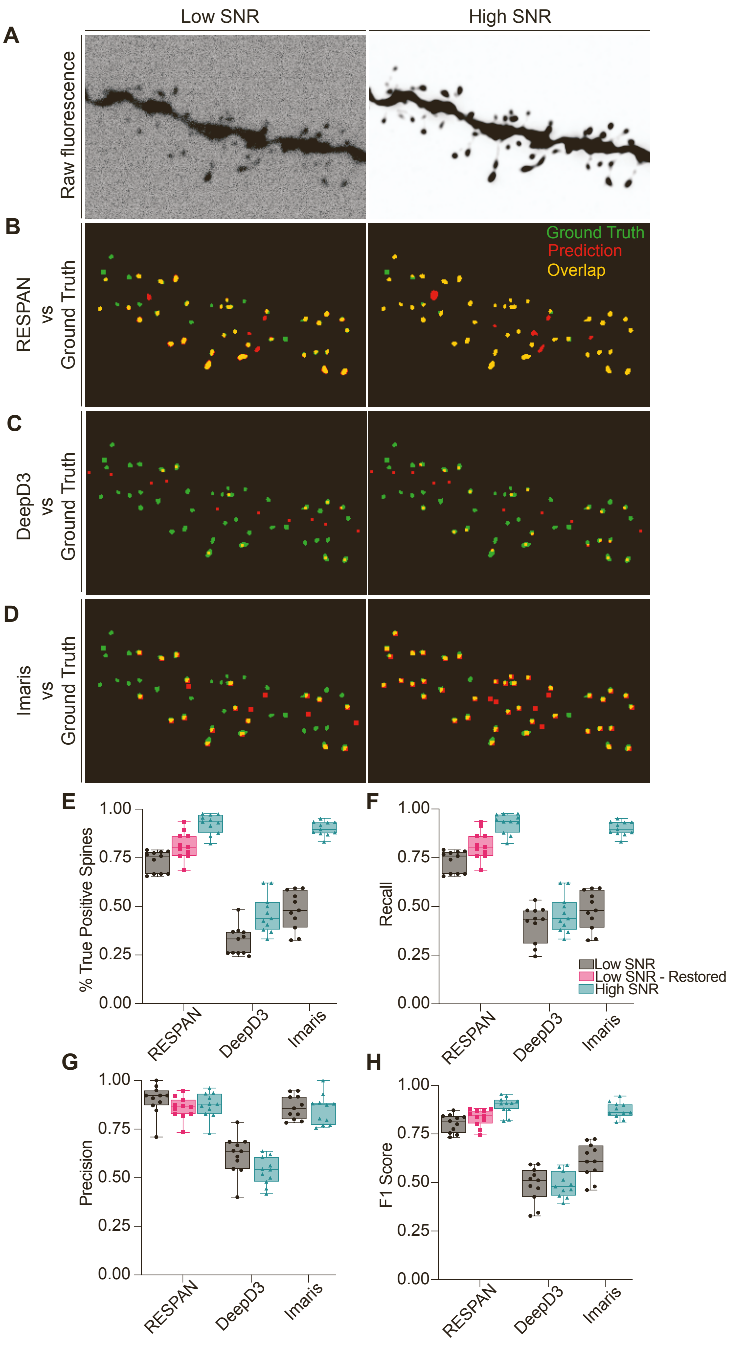

Figure S5. RESPAN outperforms DeepD3 and Imaris in spine detection across low- and high-SNR conditions. Related to Figures 2 and 3.

(A) Representative paired low-SNR and high-SNR raw fluorescence images of the same dendritic segment. (B) Overlay of RESPAN predictions (red) with expert GT labels (green); correctly overlapping voxels appear yellow. (C) DeepD3 predictions (centroids, red) and GT labels (green). (D) Spine head centroids detected by Imaris (red) and GT labels (green). (E) Percentage of true-positive spines detected: RESPAN is plotted for low-SNR, low-SN-Restored, and high-SNR images, whereas DeepD3 and Imaris are plotted for low- and high-SNR only as no integrated restoration capability is available for these tools. (F) Recall, (G) Precision, and (H) F1-score for the same conditions as in (E). Complete statistical comparisons (n = 440 analyzed spines in 3D) are provided. Scale bar, 2 μm.

Supplementary Tables

Table S1. Individual spine measurements. Related to STAR Methods.

Column	Title	Contents
1	spine_id	Unique identifier for each detected spine.
2	x	X-coordinate of the spine's centroid in image space (pixels).
3	y	Y-coordinate of the spine's centroid in image space (pixels).
4	z	Z-coordinate of the spine's centroid in image space (pixels).
5	dendrite_id	Identifier for the dendrite associated with the spine.
6	geodesic_dist_to_soma	Geodesic distance from the spine to the soma (µm).
7	euclidean_dist_to_soma	Direct/Euclidean distance between the spine head and the soma (µm).
8	spine_area	Cross-sectional area of the entire spine (µm²).
9	spine_vol	Volume of the entire spine (µm³).
10	spine_surf_area	Spine surface area adjusted for voxel resolution (µm²).
11	spine_length	Total length of the spine from the dendrite base to the spine head tip (µm).
12	spine_length_euclidean	Direct/Euclidean distance between the distal tip of the spine and the dendrite (µm).
13	spine_bbox_vol	Volume of the bounding box containing the spine (µm³).
14	spine_extent	Ratio of the volume of the spine to the volume of its bounding box.
15	spine_solidity	Ratio of the volume of the spine to the volume of its convex hull.
16	spine_convex_vol	Volume of the smallest convex polygon that can contain the spine (µm³).
17	head_area	Cross-sectional area of the spine head (µm²).
18	head_vol	Volume of the spine head (µm³).
19	head_surf_area	Surface area of the spine head adjusted for voxel resolution (µm²).
20	head_length	Length of the spine head, measured from the neck boundary to the head tip (µm).
21	head_euclidean_dist_to_den d	Direct/Euclidean distance between the spine head and the dendrite (µm).
22	head_bbox_vol	Volume of the bounding box containing the spine head (µm³).
23	head_extent	Ratio of the volume of the spine head to the volume of its bounding box.
24	head_solidity	Ratio of the volume of the spine head to the volume of its convex hull.
25	head_convex_vol	Volume of the smallest convex polygon that can contain the spine head (µm³).
26	head_convex_hull_ratio	The relative volume difference between the spine head's convex hull and actual volume. Calcuated using (Spine head convex hull volume - spine head volume) / spine head volume.
27	neck_area	Cross-sectional area of the spine neck (µm²).

28	neck_vol	Volume of the spine neck (μm³).
29	neck_surf_area	Surface area of the spine neck adjusted for voxel resolution (μm²).
30	neck_length	Length of the spine neck, measured from the dendrite to the boundary of the spine head (μm).
31	neck_bbox_vol	Volume of the bounding box containing the spine neck (μm³).
32	neck_extent	Ratio of the volume of the spine neck to the volume of its bounding box.
33	neck_solidity	Ratio of the volume of the spine neck to the volume of its convex hull.
34	neck_convex_vol	Volume of the smallest convex polygon that can contain the spine neck (μm³).
35	spine_Cn_mean_int	Mean fluorescence intensity in channel n within the entire spine.
36	spine_Cn_max_int	Maximum fluorescence intensity in channel n within the entire spine.
37	spine_Cn_int_density	The integrated density of the entire spine for channel n. Calculated by multiplying the mean fluorescence intensity in channel n by the spine volume.
38	head_Cn_mean_int	Mean fluorescence intensity in channel n within the spine head.
39	head_Cn_max_int	Maximum fluorescence intensity in channel n within the spine head.
40	head_Cn_int_density	The integrated density of the spine head for channel n. Calculated by multiplying the mean fluorescence intensity in channel n by the spine head volume.
41	neck_Cn_mean_int	Mean fluorescence intensity in channel n within the spine neck.
42	neck_Cn_max_int	Maximum fluorescence intensity in channel n within the spine neck.
43	neck_Cn_int_density	The integrated density of the spine neck for channel n. Calculated by multiplying the mean fluorescence intensity in channel n by the spine neck volume.

Table S2. Measurement Summaries (per dendritic branch and image). Related to STAR Methods.

Column	Title	Contents
1	Filename	Name of the analyzed image file.
2	res_XY	Lateral resolution of the image (µm/pixel).
3	res_Z	Axial resolution of the image (µm/pixel).
4	dendrite_length	Total length of the dendrites (µm).
5	dendrite_vol	Total dendritic volume (µm³).
6	total_spines	Total number of detected spines.
7	spines_per_um	Number of spines per micrometer of dendrite length (spines/µm).
8	spines_per_um3	Number of spines per cubic micrometer of dendrite volume (spines/µm³).
9	avg_geodesic_dist_to_soma	Mean spine geodesic distance from soma (µm).
10	avg_euclidean_dist_to_soma	Mean spine Euclidean distance from soma (µm).
11	avg_spine_area	Mean cross-sectional area of detected spines (µm²).
12	avg_spine_vol	Mean volume of detected spines (µm³).
13	avg_spine_surf_area	Mean surface area of detected spines (µm²).
14	avg_spine_length	Mean length of detected spines (µm).
15	avg_spine_length_euclidean	Mean direct distance between the distal tip of the spine and the dendrite (µm).
16	avg_spine_bbox_vol	Mean volume of the bounding box of detected spines (µm³).
17	avg_spine_extent	Mean extend of detected spines.
18	avg_spine_solidity	Mean solidity of detected spines.
19	avg_spine_convex_vol	Mean convex volume of detected spines µm²).
20	avg_head_area	Mean cross-sectional area of detected spine heads (µm²).
21	avg_head_vol	Mean volume of detected spine heads (µm³).
22	avg_head_surf_area	Mean surface area of detected spine heads (µm²).
23	avg_head_length	Mean length of detected spines heads (µm).
24	avg_head_euclidean_dist_to_de nd	Mean Direct/Euclidean distance between the spine heads and the dendrite (µm).
25	avg_head_bbox_vol	Mean volume of the bounding box of detected spine heads (µm³).
26	avg_head_extent	Mean extend of detected spine heads.
27	avg_head_solidity	Mean solidity of detected spine heads.
28	avg_head_convex_vol	Mean convex volume of detected spine heads (µm²).

29	avg_head_convex_hull_ratio	Mean convex hull ratio of detected spine heads.
30	avg_neck_area	Mean cross-sectional area of detected spine necks (μm^2).
31	avg_neck_vol	Mean volume of detected spine necks (μm^3).
32	avg_neck_surf_area	Mean surface area of detected spine necks (μm^2).
33	avg_neck_length	Mean length of detected spine necks (μm).
34	avg_neck_bbox_vol	Mean volume of the bounding box of detected spine necks (μm^3).
35	avg_neck_extent	Mean extend of detected spine necks.
36	avg_neck_solidity	Mean solidity of detected spine necks.
37	avg_neck_convex_vol	Mean convex volume of detected spine necks (μm^2).
38	avg_spine_C1_mean_int	Mean fluorescence intensity in channel n within the spines.
39	avg_spine_C1_max_int	Mean maximum fluorescence intensity in channel n within the spines.
40	avg_spine_C1_int_density	Mean integrated density of the spines for channel n.
41	avg_head_C1_mean_int	Mean fluorescence intensity in channel n within the spine heads.
42	avg_head_C1_max_int	Mean maximum fluorescence intensity in channel n within the spine heads.
43	avg_head_C1_int_density	Mean integrated density of the spine heads for channel n.
44	avg_neck_C1_mean_int	Mean fluorescence intensity in channel n within the spine necks.
45	avg_neck_C1_max_int	Mean maximum fluorescence intensity in channel n within the spine necks.
46	avg_neck_C1_int_density	Mean integrated density of the spine necks for channel n.

Table S3. Measurement summary (temporal analysis). Related to STAR Methods.

Column	Title	Contents
1	timepoint	Timepoint of the recorded data.
2	res_XY	Lateral resolution (µm/pixel).
3	res_Z	Axial resolution (µm/pixel).
4	dendrite_length	Total length of the segmented dendrite (µm).
5	dendrite_vol	Total dendritic volume (μm^3).
6	total_spines	Total number of detected spines at this timepoint.
7	spines_per_um	Density of spines per unit dendritic length (spines/µm).
8	new_spines	Number of newly detected spines at this timepoint.
9	pruned_spines	Number of spines that disappeared compared to the previous timepoint.
10	spines_per_um3	Density of spines per unit dendritic volume (spines/μm^3).
11	avg_spine_area	Mean spine cross-sectional area (μm^2).
12	avg_spine_area_um2	Mean spine cross-sectional area (μm^2).
13	avg_spine_vol	Mean spine volume (μm^3).
14	avg_C1_mean_int	Mean fluorescence intensity in channel 1 within spines.
15	avg_spine_length	Mean spine length (µm).
16	avg_C1_max_int	Maximum fluorescence intensity in channel 1 within spines.
17	avg_dist_to_dendrite	Mean geodesic distance of spines to the dendrite (µm).
18	avg_dist_to_soma	Mean geodesic distance of spines to the soma (µm).
19	avg_C1_int_density	Mean fluorescence intensity density in channel 1 within spines.

Table S4. Segmentation validation. Related to STAR Methods.

Column	Title	Contents
1	Filename	Input image filename.
2	res_XY	Lateral image resolution (µm/pixel).
3	res_Z	Axial image resolution (µm/pixel).
4	gt_total_spines	Total spines in the ground truth image.
5	gt_total_spines_filtered	Total spines analyzed post-filtering in the ground truth image.
6	output_total_spines	Total spines detected by RESPAN.
7	output_total_spines_filtered	Total spines analyzed post-filtering by RESPAN.
8	TruePos_IoU50	True positive spines detected.
9	FalsePos_IoU50	False positive spines detected.
10	FalseNeg_IoU50	False negative spines.
11	Spine_precision_IoU50	Precision of spine detection.
12	Spine_recall_IoU50	Recall of spine detection.
13	gt_total_spine_vol	Total spine volume in the ground truth image (μm^3).
14	output_total_spine_vol	Total spine volume detected by RESPAN (μm^3).
15	total_spine_iou	IoU of spine voxels between ground truth and RESPAN.
16	total_spine_vol_difference	Difference in total spine volume between ground truth and RESPAN (µm).
17	gt_dendrite_length	Total dendritic length in the ground truth image (µm).
18	output_dendrite_length	Total dendritic length detected by RESPAN (µm).
19	dendrite_length_difference	Difference in dendritic length between ground truth and RESPAN (µm).
20	gt_dendrite_vol	Total dendritic volume in the ground truth image (μm^3).
21	output_dendrite_vol	Total dendritic volume detected by RESPAN (μm^3).
22	total_dendrite_iou	IoU of dendrite voxels between ground truth and RESPAN.
23	dendrite_vol_difference	Difference in dendritic volume between ground truth and RESPAN (μm^3).

Table S5. Comparison of existing software tools for spine and dendrite analysis. Related to STAR Methods.

Tool	RESPAN	SpineTool	DeepD3	3DSpan	DeepSpine Tool	Imaris
**Compatible Input Formats**	.tif	.tif	.tif	.tif, .hdr, .img	.tif	.ims
**Machine Learning Capabilities**	Image restoration (SNR, resolution), Segmentation (2D/3D U-Net)	–	Segmentation (2D U-Net)	–	Segmentatio n (2D U-Net)	Segmentation (pixel classification)
**Image Outputs**	Spine, dendrite, soma masks (.tif), 2D/3D spine arrays (.tif), dendrite tracing (.swc)	Binarized spine image (.tif), spine meshes (.off)	Spine ROIs (.rois), spine masks (.tiff), prediction maps (.prediction)	Segmented sub-volumes (.hdr/.img)	Segmentatio n mask (.tif)	Objects visible within Imaris and can be converted to image data for export
**Tabular Outputs**	Spine counts and metrics (.csv)	Spine/dendrite classification (.json)	None	Segmentation analysis (.csv)	None	Parameters can be manually exported (.csv/.xlsx)
**Installation Requirements**	Windows, NVIDIA GPU, Anaconda	Windows, 1GB RAM, Anaconda	Python 3.7, Anaconda, TensorFlow	Windows	Windows, Python 3.6, CUDA 10.1, 8GB GPU	Windows/Mac
**Training Data Acquisition Modality**	2-photon, spinning disk confocal, Airyscan	Confocal	2-photon	2-photon	–	–
**User Interface**	GUI (single/batch processing)	Jupyter Notebook	GUI (single image, scripting for batch)	GUI	GUI	GUI
**Potential Limitations**	Training of data specific models	Sensitive to SNR and image quality	Training of data specific models and parameter tuning	Sensitive to SNR and image quality	Training of data specific models	Sensitive to SNR and image quality
**Open Source**	Yes	Yes	Yes	Yes	No	No
**Batch Processing**	Yes	No	Yes	No	No	Yes
**User-Trainable Models**	Yes	No	Yes	No	No	No
**Spine Quantification**	Yes	No	Yes	Yes	No	Yes
**Validation Capability**	Yes	No	Yes	No	No	No
**ImageJ Compatibility**	Yes	Yes	Yes	No	Yes	No
